# Membrane Processes for Remediating Water from Sugar Production By-Product Stream

**DOI:** 10.3390/membranes15070207

**Published:** 2025-07-12

**Authors:** Amal El Gohary Ahmed, Christian Jordan, Eva Walcher, Selma Kuloglija, Reinhard Turetschek, Antonie Lozar, Daniela Tomasetig, Michael Harasek

**Affiliations:** 1 Institute of Chemical Environmental & Bioscience Engineering E166, Technische Universität Wien, 1060 Vienna, Austria; eva.m.walcher@tuwien.ac.at (E.W.); selma.kuloglija@tuwien.ac.at (S.K.); michael.harasek@tuwien.ac.at (M.H.); 2 AGRANA Research & Innovation Center GmbH, Josef-Reither-Strasse 21-23, 3430 Tulln, Austria; reinhard.turetschek@agrana.com (R.T.); antonie.lozar@students.boku.ac.at (A.L.); 3 Institute of Chemical Technologies and Analytics E164, Technische Universität Wien, 1060 Vienna, Austria; daniela.tomasetig@tuwien.ac.at

**Keywords:** sugar production by-product streams, membrane processes, water treatment, water quality

## Abstract

Sugar production generates wastewater rich in dissolved solids and organic matter, and improper disposal poses severe environmental risks, exacerbates water scarcity, and creates regulatory challenges. Conventional treatment methods, such as evaporation and chemical precipitation, are energy-intensive and often ineffective at removing fine particulates and dissolved impurities. This study evaluates membrane-based separation as a sustainable alternative for water reclamation and sugar recovery from sugar industry effluents, focusing on replacing evaporation with membrane processes, ensuring high permeate quality, and mitigating membrane fouling. Cross-flow filtration experiments were conducted on a lab-scale membrane system at 70 °C to suppress microbial growth, comparing direct reverse osmosis (RO) of the raw effluent to an integrated ultrafiltration (UF)–RO process. Direct RO resulted in rapid membrane fouling. A tight UF (5 kDa) pre-treatment before RO significantly mitigated fouling and improved performance, enabling 28% water recovery and 79% sugar recovery, maintaining permeate conductivity below 0.5 mS/cm, sustaining stable flux, and reducing membrane blocking. Additionally, the UF and RO membranes were tested via SEM, EDS, and FTIR to elucidate the fouling mechanisms.

## 1. Introduction

Water plays a pivotal role in the beet sugar industry, being indispensable across multiple stages of production, including sugar beet washing, juice extraction, purification, evaporation, crystallization, and equipment cleaning [1,2]. However, the industry is also a significant generator of wastewater, often referred to as raffinate or by-product streams, which contain high concentrations of organic matter, residual sugars, and dissolved salts [3,4]. Improper disposal of these effluents can result in elevated chemical oxygen demand (COD), biological oxygen demand (BOD), and total dissolved solids (TDS), leading to severe pollution of receiving water bodies [5,6]. In addition, increasing global concerns regarding water scarcity and environmental pollution have placed the sugar industry under mounting pressure to implement more sustainable water management and wastewater treatment solutions [7,8].

Conventional treatment methods in the sugar sector, such as sedimentation, chemical coagulation, evaporation, and biological treatment, have been widely employed [9]. However, these methods typically suffer from limited efficiency in removing dissolved organic matter and fine particulates, high energy consumption, and suboptimal water recovery [10,11]. Consequently, there is growing interest in membrane-based separation processes as an alternative for advanced wastewater treatment and high-quality water reclamation [12,13,14].

Membrane filtration technologies such as ultrafiltration (UF), nanofiltration (NF), and reverse osmosis (RO) are increasingly recognized for their ability to produce permeate of superior quality while offering modularity, scalability, and reduced chemical usage [15,16]. Reverse osmosis, in particular, provides high rejection rates of dissolved salts, sugars, and organic contaminants, making it well-suited for reclaiming water from sugar processing effluents [17,18,19]. However, when treating raffinate streams directly, RO membranes are prone to rapid fouling due to the accumulation of organic matter, scaling, biofouling, and colloidal deposition [20,21]. Such fouling not only compromises membrane performance but also leads to increased operational costs, frequent cleaning, and shortened membrane lifespan.

To address these challenges, pre-treatment using UF is widely employed to remove suspended solids, colloidal particles, and high molecular weight organic compounds prior to RO [22,23]. UF membranes with tight molecular weight cut-offs (such as 5 kDa) have demonstrated significant potential in reducing the fouling propensity of RO membranes, improving overall system efficiency, and enabling sustainable long-term operation [24].

Moreover, understanding the nature and distribution of membrane fouling is critical for optimizing membrane processes in industrial applications. Advanced characterization techniques such as Fourier-transform infrared spectroscopy (FTIR), Raman spectroscopy, energy-dispersive X-ray spectroscopy (EDX), and scanning electron microscopy (SEM) offer powerful tools for identifying foulant composition and spatial distribution along membrane modules [25,26,27,28]. Evaluating fouling at different locations within the membrane element—namely, the entrance, middle, and end—provides valuable insights into fouling mechanisms and informs the development of improved system design and cleaning protocols [29].

This study investigates a combined membrane filtration approach for recovering high-quality water from beet sugar industry raffinate streams. Specifically, a two-stage process comprising tight ultrafiltration (UF, 5 kDa cut-off) followed by reverse osmosis (RO) is employed to overcome the limitations of direct RO treatment. Furthermore, the study systematically characterizes membrane fouling along the membrane length using FTIR, Raman spectroscopy, EDX, and SEM to elucidate fouling mechanisms and enhance process performance for sustainable water reuse in sugar production.

## 2. Materials and Methods

### 2.1. Feed Composition

**Figure 1:**
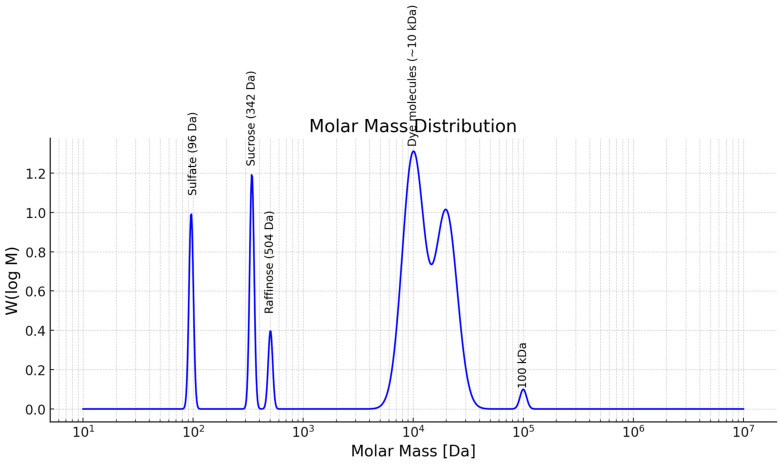
Size exclusion chromatography (SEC) analysis of the by-product stream from the beet sugar industry.

The by-product stream from the beet sugar industry was sourced from an Austrian sugar refinery. This feed solution exhibits a high conductivity of approximately 18 mS/cm and has a concentration of 1.5 °Brix. The stream contains raffinose, sucrose, and sulphate, and has a dark brown color (Table 1). The size exclusion chromatography (SEC) profile of the stream is illustrated in Figure 1.

### 2.2. Experimental Setup and Procedure

The by-product stream was treated using an integrated membrane process. Initially, the stream passed through a UF membrane from Alfa Laval with a molecular weight cutoff (MWCO) of 5 kDa. The permeate from this stage was then processed through an RO membrane supplied by Alfa Laval. These membranes were capable of operating at temperatures of up to 75 °C. The characteristics of the two membranes are detailed in Table 2.

**Figure 2:**
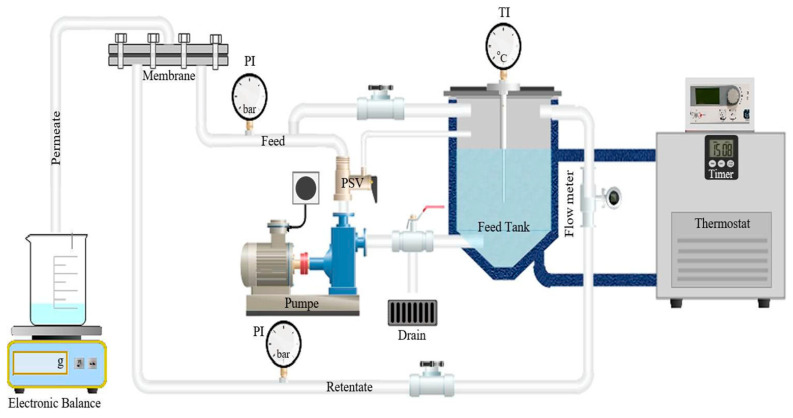
Schema of membrane filtration unit (model OS-MC-01) [30].

The experiments were conducted at laboratory scale using a cross-flow membrane filtration system from Osmo (Münster, Germany), model OS-MC-01 (see Figure 2). The system includes a 2 L stainless steel jacketed feed tank. The feed solution is directed into a rectangular flat sheet cross-flow membrane module with an effective membrane area of 0.008 m^2^ (dimensions: 0.04 m by 0.2 m). This process is powered by a CAT high-pressure piston pump, model 231, which can deliver a 3.7 L/min flow rate and achieve pressures of up to 60 bar. Throughout the experiments, the setup operated in batch concentration mode, wherein the retentate was recirculated back into the feed tank while the permeate was continuously discharged from the system.

Both UF and RO membranes were soaked in DI water for 2 h to ensure thorough hydration and achieve optimal swelling. After that, they were subjected to high-pressure compaction for 30 min to enhance their structural integrity and mechanical properties.

The water permeability was assessed both before and after the filtration process to determine the degree of membrane fouling.

All experiments were conducted at 70 °C to inhibit microbial growth in the feed solution and at a fixed cross-flow of around 3.7 L/min. The operating pressure for the UF process was 8 bar, while for the RO process, it was set at 32 bar.

Two experimental configurations were examined. In the first experiment, the feed stream was treated directly with the reverse osmosis (RO) membrane without any prior treatment. In the second experiment, the feed stream was first subjected to ultrafiltration (UF), and then the UF permeate was treated with the RO membrane. Each experiment was operated until a recovery of 70% was achieved. The permeate flow was recorded, and samples were collected from the feed before treatment, as well as from the permeate and retentate, for further analysis to determine the performance of the process.

### 2.3. Analysis

The conductivity of the feed, retentate, and permeate streams was measured to assess the rejection of salts. Conductivity was measured with a WTW TetraCon 925 (Xylem, Weilheim, Germany) conductivity probe coupled to a WTW Multi 3430 (Xylem, Weilheim, Germany). Ion chromatography was performed on a Dionex ICS-5000 system (Thermo Scientific, California, CA, USA) and was employed to determine the rejection of anions and cations.

To determine the quantities of sucrose and raffinose, samples were collected from each experimental stage: feed, permeate, and retentate. These samples were subsequently analyzed using high-performance liquid chromatography (HPLC) (Agilent Technologies, Santa Clara, CA, USA). For all measurements, the HPLC method utilized a Shimadzu UFLC system; the analytical flow rate was adjusted to 0.6 mL/min, and the injection volume was 10 µL. The mobile phase was 5 mM of H_2_SO_4_, and the analysis was performed under isocratic conditions. The temperature was maintained at 50 °C. Detection was carried out using a RID-10A refractive index detector. The separation was achieved using a Shodex SH1011 column (8 × 300 mm), accompanied by an SH-G Sugar guard column.

Fourier transform infrared (FTIR) spectroscopy was used to investigate membrane fouling and identify surface functional groups on both fresh and used RO and UF membranes. For the used membranes, samples were taken from three locations along the module—entrance (0–5 cm), middle (5–15 cm) and end (15–20 cm). To investigate the phenomenon of membrane fouling and to determine surface functional groups present on the membrane, the fresh RO and UF membrane and used RO and UF membrane at different positions of the membrane (entrance (0–5 cm), middle (5–15 cm), and end (15–20 cm)) were tested by Fourier Transform Infrared spectroscopy (FTIR) (Shimadzu, Kyoto, Japan). FTIR spectral data were collected using a BRUKER VERTEX 70 (Billerica, MA, USA) spectrometer, which enabled measurements over a wavelength range of 4000 to 400 cm^−1^. To improve the signal quality, a total of 64 individual scans were performed and averaged, with a spectral resolution set at 2 cm^−1^.

Raman spectroscopy (Witec Alpha 300RSA device) (Ulm, Germany) was used to analyze fouling on the reverse osmosis (RO) membrane. Due to the high surface roughness and opacity of the active layer, Raman measurements could not be performed from the feed side; therefore, spectra were collected from the permeate (back) side of the membrane. Raman spectroscopy provides complementary information to Fourier Transform Infrared (FTIR) spectroscopy, as it relies on inelastic light scattering rather than infrared absorption, making it particularly sensitive to non-polar bonds and crystalline phases. Additionally, Raman spectroscopy can probe through semi-transparent materials, enabling effective analysis from the membrane’s reverse side, where FTIR is limited by penetration depth and sample geometry.

To characterize the extent and composition of fouling on both RO and UF membranes along the length of the membrane (at the same different positions), Scanning Electron Microscopy (SEM) and Energy-Dispersive X-ray Spectroscopy (EDX) were employed. A FEI Quanta FEG250 microscope (Hillsboro, OR, USA) was used to perform SEM imaging. Membrane samples were mounted on aluminum stubs with a carbon adhesive tape. Also, a Q150T S sputter coater was used to coat the membrane samples with a thin layer of gold, operated at 15 mA for 5–10 min to improve surface conductivity. Elemental analysis of the fouling layers was subsequently conducted using EDX on the same SEM instrument (Hitachi High-Technologies, Tokyo, Japan), providing complementary information on the inorganic and organic components present on the membrane surfaces.

### 2.4. Performance Metrics

#### 2.4.1. Flux (*J*)

Flux (*J*) in membrane processes is the rate at which the permeate passes through the membrane per unit membrane area and per unit time.

(1) $$J=\frac{∆m}{A\;t}\;\;\;\;\;\;\;\;(\frac{kg}{m^{2}.h})$$

∆m = mass of permeate collected over time, kg, *A* = effective membrane area m^2^
*t* = time h

#### 2.4.2. Rejection, *R*

Rejection is the percentage of solute that is retained by the membrane rather than permeating through it. The rejection (*R*) was determined using Equation (1), where *C_p_* represents the permeate concentration and *C_F_* denotes the feed concentration.

(2) $$R\%=\left(1-\left(\frac{C_{p}}{C_{F}}\right)\right)*100$$

#### 2.4.3. The Flux Recovery Ratio (*FRR*)

The Flux Recovery Ratio (*FRR*) is a simple way to quantify how well your membrane regains its pure-water permeability after fouling and subsequent cleaning. It is defined as the ratio of the cleaned membrane’s pure-water flux to its original (pristine) pure-water flux, usually expressed as a percentage:

(3) $$The\;Flux\;Recovery\;Ratio\;\left(FRR\right)=\frac{J_{w,\;after\;cleaning}}{J_{w,\;Intial}}\times 100\%$$

## 3. Results and Discussion

Initial direct RO trials performed on the beet-sugar raffinate feed under constant-pressure (32 bar) and ambient-temperature (25 °C) conditions exhibited a rapid and irreversible decline in permeate flux, culminating in complete membrane blockage and cessation of permeate passage. Throughout each 2 h run, the water permeability coefficient decreased by 78% of its original value, while the permeate flux dropped precipitously from an initial 30 kg/m^2^·h to below 4 kg/m^2^·h. Standard chemical cleaning procedures—including a 30 min soak in 0.1 M HCl (pH ≈ 2) followed by a 0.1 M NaOH rinse (pH ≈ 12)—failed to restore membrane performance, suggesting that irreversible fouling or pore blockage by organic macromolecules had occurred. In light of these results, we developed an integrated treatment sequence in which the raffinate was first subjected to UF to remove colloidal and high-molecular-weight foulants prior to RO. The performance of this UF–RO integrated system, in terms of permeate flux stabilization, fouling mitigation, and solute rejection, is detailed in the following section.

### 3.1. Separation Performance of the Integrated UF–RO Process

#### 3.1.1. SEC of UF Membrane (Feed–Permeate–Retentate)

**Figure 3:**
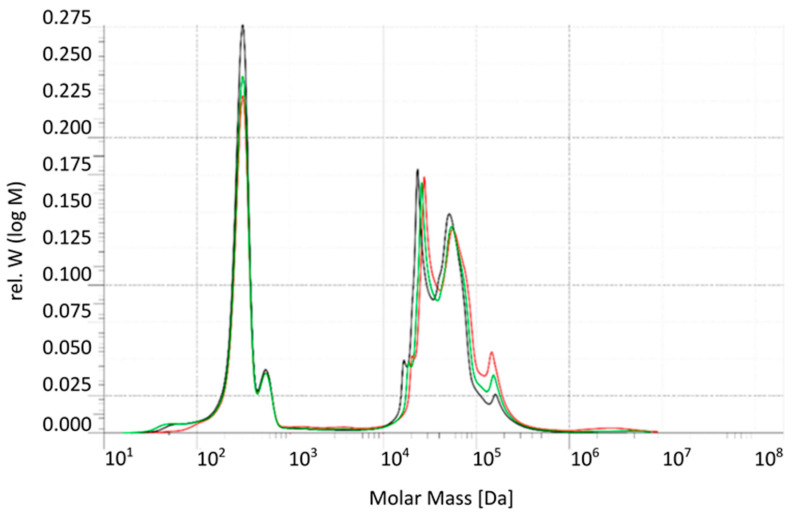
SEC of UF membrane (Feed–Permeate–Retentate)—green line: feed, red line: retentate, black line: permeate.

Size Exclusion Chromatography (SEC) was performed to evaluate the molecular weight distribution of organic compounds in the raffinate stream from the beet sugar industry following treatment with a 5 kDa UF membrane (see Figure 3). The molar mass distribution (M) on the x-axis is given on a logarithmic scale. The y-axis shows the relative intensity of the analyte signal at a given log of the molecular weight, denoted as w (log M), to ensure that the integral under the curve is normalized. The analysis compared the feed (green curve), permeate (black curve), and retentate (red curve) streams. In the low-molecular-weight range (~10^2^–10^3^ Da), corresponding primarily to simple sugars (e.g., glucose, fructose, sucrose fragments), the permeate and feed profiles were nearly identical, indicating that these compounds readily passed through the UF membrane.

The intermediate molecular weight region (~10^4^–10^5^ Da) is likely associated with oligosaccharides, glycoproteins, and melanoidins. The high-molecular-weight region (>10^5^ Da) includes polysaccharides, protein aggregates, and other potential foulants of the membrane.

It appears that all three curves overlay almost perfectly across the entire molar-mass range except at the peak near 10^2^ Da and above 10^5^ Da. At the peak at 10^2^ Da, the black permeate trace falls noticeably above the retentate, indicating increased permeance only at that low-molecular-weight region. In contrast, above 10^5^ Da, the permeate is significantly depleted of analyte, which can be found in the retentate (red line).

These findings confirm that the 5 kDa UF membrane effectively separates high- and intermediate-molecular-weight foulants from low-molecular-weight sugars, providing a helpful pre-treatment step to reduce fouling potential in subsequent membrane processes such as RO.

#### 3.1.2. SEC of RO Membrane (Feed–Permeate–Retentate)

**Figure 4:**
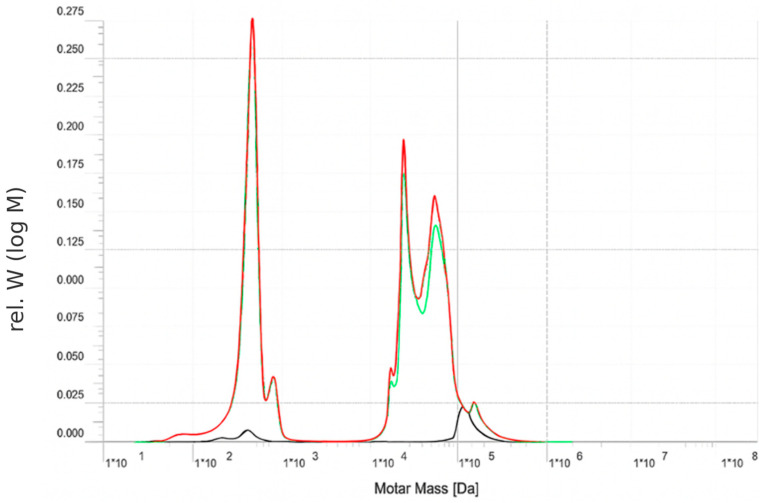
Size exclusion chromatography (SEC) of RO membrane (Feed–Permeate–Retentate)—green line: feed, black line: permeate, red line: retentate. The peak of RO permeate (black line) at 10^5^ Da is unexpected and needs further investigation.

The separation and purification of water using an RO membrane after pre-treatment of the by-product stream with a UF membrane are analyzed through size exclusion chromatography (SEC). In this process, only water and monovalent ions pass to the permeate side, resulting in a conductivity of approximately 0.462 mS/cm. The SEC results for the permeate, retentate, and feed are illustrated in Figure 4.

The green curve, representing the feed, exhibits a bimodal distribution. The first peak, observed in the molar mass range of approximately 10^2^ to 10^3^ Da, corresponds to the presence of low molecular weight sugars, specifically sucrose (342 Da) and raffinose (504 Da). The second major peak, appearing near 10^4^ Da, is attributed to higher molecular weight species, including brown-colored compounds and proteins typically present in beet sugar processing by-products.

The red curve, corresponding to the retentate, closely mirrors the feed profile in the higher molar mass region (~10^4^ Da), indicating effective retention of proteins and brown-colored compounds by the RO membrane.

The black curve, representing the permeate, shows a low overall signal, with a small peak evident below 10^2^–10^3^ Da. This indicates that a portion of the low molecular weight sugars, such as sucrose and raffinose, is able to pass through the membrane. The extra peak at 10^5^ Da is unexpected and will require follow-up.

The observed data confirms that the RO membrane is performing as expected for this application. High molecular weight species, which contribute to color and fouling potential, are effectively retained. Partial passage of sucrose and raffinose through the membrane is characteristic of RO operation, as complete rejection of small neutral sugars is challenging even for tight membranes.

In summary, the RO process applied to this beet sugar by-product stream achieves excellent removal of high molecular weight contaminants while allowing partial transmission of desirable sugars. The current membrane performance appears well-suited for applications aiming at decolorization or purification of the feed stream. Further optimization, such as selection of higher rejection RO membranes or adjustment of operating parameters, may be considered if the goal is to maximize sugar retention or water purity.

#### 3.1.3. UF Rejection Performance

**Figure 5:**
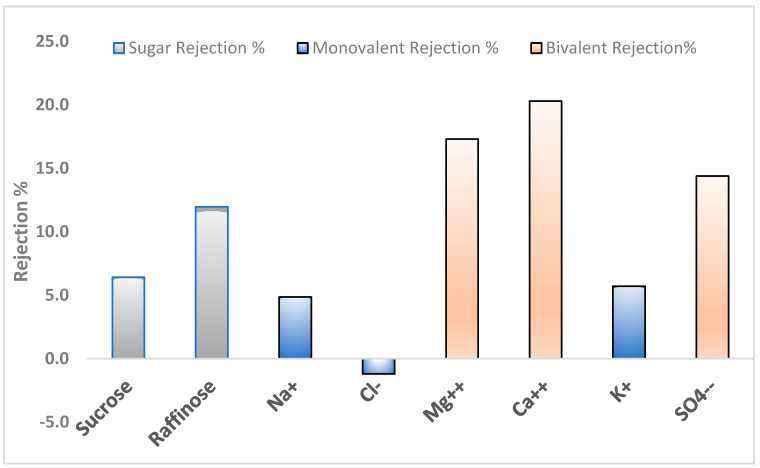
Rejection of sugars and monovalent and bivalent ions by UF membrane.

The rejection performance of the UF GR90 PP membrane with a 5 kDa (MWCO) was evaluated for various solutes, including sugars and inorganic ions, as summarized in Figure 5.

The rejection (*R*) was determined using Equation (2), where *C_p_* represents the permeate concentration and *C_F_* denotes the feed concentration.

The membrane demonstrated substantial rejection of neutral sugars, with 6.4% rejection for sucrose (molecular weight ~342 Da) and 12.0% for raffinose (~504 Da), despite both being smaller than the nominal MWCO. This indicates that the membrane may exhibit an effective MWCO lower than 5 kDa or that additional factors such as steric hindrance, solute shape, and hydrophilic–hydrophobic interactions influence transport.

Furthermore, no rejection was observed for monovalent ions, given that UF membranes are generally not designed for salt separation. The rejection of bivalent ions was also noteworthy: Mg^2+^ and Ca^2+^ were rejected at 17.3% and 20.3%, respectively, consistent with the known tendency of bivalent ions with larger hydration shells to experience greater steric and electrostatic repulsion. The SO_4_^2−^ rejection was 15%

Overall, the observed performance indicates that the UF GR90 PP membrane operates with a combination of size-based and electrostatic exclusion mechanisms. It is also possible that membrane surface properties, fouling, or operational conditions (e.g., pH, ionic strength) contributed to the higher-than-expected ion rejection. These findings suggest that this membrane, although classified as ultrafiltration, displays behaviors partially characteristic of tight UF or loose nanofiltration (NF) membranes.

### 3.2. Qualitative and Quantitative Fouling Analysis

#### 3.2.1. FTIR of the UF Membrane (GR90PP, 5 kDa)

**Figure 6:**
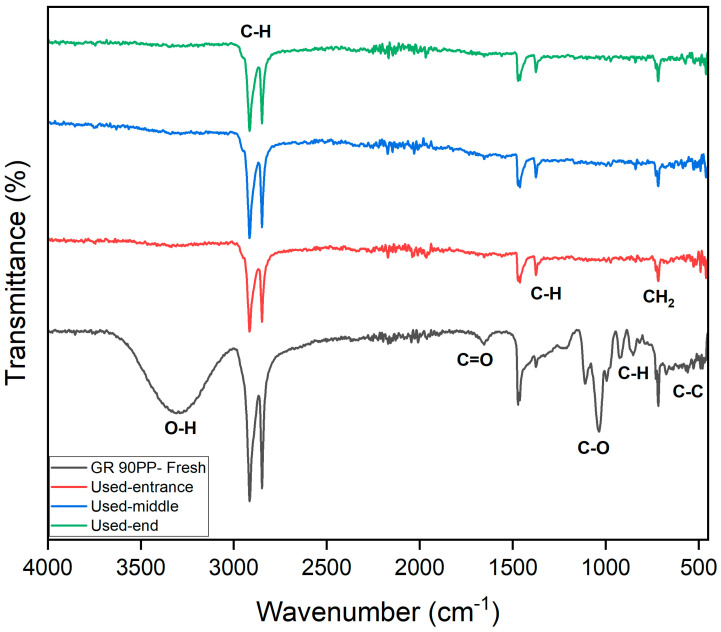
FITR of UF membrane.

Figure 6 represents the FTIR of the UF membrane (GR90PP, 5 kDa), which reveals notable changes in membrane chemistry after use, providing insights into the fouling behavior during treatment of the raffinate solution. In the fresh membrane, the prominent broad O–H/N–H stretching band (~3600–3200 cm^−1^) and the sharp C–H stretching band (~2920–2850 cm^−1^) are clearly visible [31]. In the used membranes, these bands show noticeable attenuation, particularly at the membrane end, which indicates surface coverage or chemical modification by fouling layers, likely composed of organic materials and biofilm components.

The Amide I and II regions (~1700–1500 cm^−1^) exhibit broadening and reduced intensity in the used samples, again suggesting the accumulation of proteinaceous substances or chemical interactions between foulants and the membrane material. The C–O–C and C–O stretching region (~1230–1020 cm^−1^), though less pronounced than in the RO membrane, also shows some increase in absorbance in the used membrane, which points to the deposition of polysaccharides or sugar-derived materials from the raffinate.

A progressive fouling pattern is evident across the membrane length. The spectra show that fouling increases from the entrance to the middle and is most pronounced at the membrane end, where concentration polarization would be most significant. Additionally, the baseline shift and increased absorbance across the entire fingerprint region suggest the formation of a heterogeneous fouling layer comprising both organic and inorganic components. UF membrane experienced significant organic fouling, dominated by polysaccharides and biofilm constituents, with contributions from proteins and potential inorganic interactions.

#### 3.2.2. FTIR of the RO Membrane

**Figure 7:**
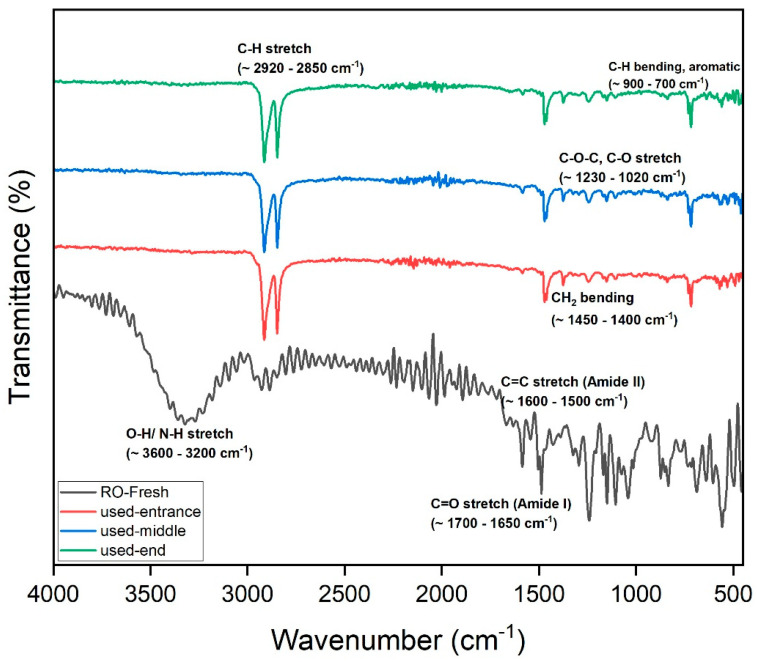
FITR of RO membrane.

The FTIR spectra provide valuable insights into the fouling behavior of the RO membrane used for treating raffinate from the beet sugar industry (Figure 7). Comparing the fresh membrane to the used membrane at various positions (entrance, middle, and end), several key changes are evident. The broad O–H/N–H stretch (~3600–3200 cm^−1^) is significantly reduced in the used membranes, suggesting the formation of a fouling layer that masks or alters these functional groups. Similarly, the C–H stretch (~2920–2850 cm^−1^) diminishes, consistent with the deposition of hydrophilic substances, such as polysaccharides and biofilm materials. In the Amide I (~1700–1650 cm^−1^) and Amide II (~1600–1500 cm^−1^) regions, peak broadening and intensity changes indicate chemical interactions with organic foulants and possible oligopeptide or protein accumulation.

Notably, the C–O–C and C–O stretch region (~1230–1020 cm^−1^) shows increased absorbance in the used membranes, a strong indicator of polysaccharide fouling. This is expected given the raffinate’s high content of residual sugars and polysaccharides, which can readily deposit on the membrane surface. Additionally, distortions and elevated absorbance in the fingerprint region (<700 cm^−1^) suggest the presence of complex inorganic-organic fouling, potentially involving metal-organic complexes or scaling components [32].

Fouling intensity increases from the entrance to the middle and is most severe at the end of the membrane. This pattern aligns with the expected effects of concentration polarization, which intensifies toward the membrane outlet, promoting greater foulant accumulation. The fouling is particularly comprised of polysaccharides and biofilm components, with contributions from proteins and inorganic species. These results highlight the need for pre-treatment and cleaning strategies to maintain the membrane.

#### 3.2.3. Raman Spectroscopy of RO Membrane (Ro 98 pHt)

**Figure 8:**
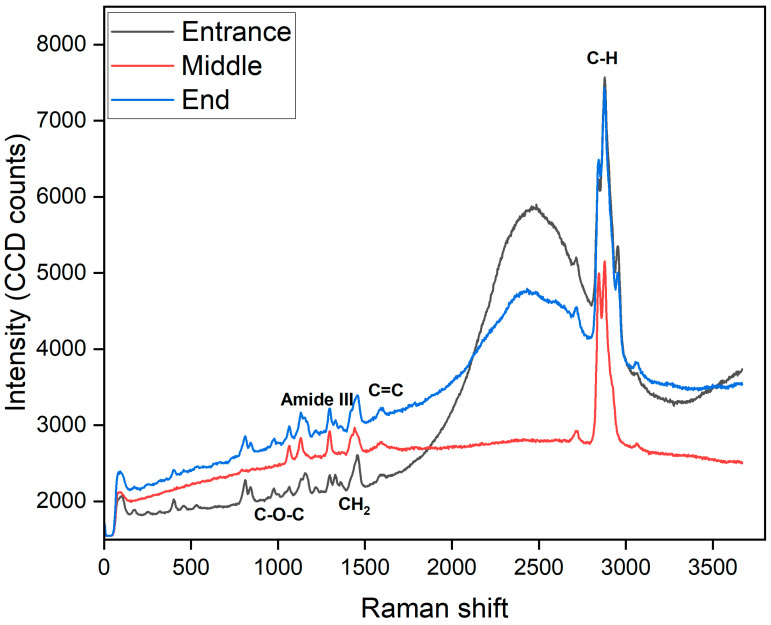
Raman spectroscopy Alfa Laval RO 98pHt membrane.

Also in this study, Raman spectroscopy was employed to analyze used Alfa Laval RO 98pHt membranes that had been operated for the treatment of by-product stream in the beet sugar industry, and these are represented in Figure 8. Given the challenging composition of this feed stream—rich in organic matter, polysaccharides, proteins, and salts—membranes used in this application are highly susceptible to various forms of fouling. The Raman spectra collected from the back side of the membranes (support layer) reveal important insights into the nature of the fouling present. In particular, the spectrum exhibits a broad, elevated baseline as well as a strong peak around 2900 cm^−1^, corresponding to C–H stretching vibrations. Such spectral features are characteristic of organic fouling and biofouling, likely originating from residual sugars, microbial extracellular polymeric substances (EPS), and proteins associated with biofilm formation. Typical Raman signatures of biofouling include bands near 1600 cm^−1^ (aromatic C=C, proteins), 1450 cm^−1^ (CH_2_ bending from lipids), 1330 cm^−1^ (amide III from proteins), and around 1000–1100 cm^−1^ (C–O–C vibrations from polysaccharides). In contrast, inorganic scaling, such as calcium carbonate or gypsum, would be identified by distinct sharp peaks at approximately 1085 cm^1^ or 980 cm^1^, which were slightly observed in this dataset, suggesting that scaling was not a dominant fouling mechanism in this case; see Figure 8.

Intense, sharp bands dominate the Raman spectrum collected at the feed-side (Entrance) at ~1100 cm^−1^ (C–O–C ether), ~1240 cm^−1^ (Amide III), ~1600 cm^−1^ (aromatic C=C), and the strong CH-stretch doublet at 2850–2950 cm^−1^—all hallmarks of the highly cross-linked aromatic–aliphatic polyamide selective layer (peak heights approaching 6–8 × 10^3^ counts). In stark contrast, the middle spectrum is essentially flat (~2.5 × 10^3^ counts) with virtually no discernible ether, amide, or aromatic features, consistent with the porous, largely aliphatic polysulfone support. At the permeate side (End), the intensities recover to intermediate values (~2.2–5 × 10^3^ counts), and both the C–O–C and Amide III bands reappear—albeit broadened and attenuated—alongside a persistent CH-stretch, reflecting the blended chemistry of residual polyamide skin and the backing layer. Together, these three spectra neatly trace the membrane’s layered architecture: a dense active film, weakly Raman-active support, and a composite backing.

In the central region of the Alfa Laval RO membrane (indicated by the red trace), there is no band observed around 2500 cm^−1^ because polyamide lacks strong fundamental vibrations in that range; instead, only very weak overtones or combination bands are present. The thinner, less crosslinked skin layer at this position leads to lower overall Raman intensity, causing these faint features to fall within the noise level. Furthermore, the rising envelope of C–H that stretches around 2900 cm^−1^ further obscures any minor peaks that might be present near 2500 cm^−1^. This aligns with the findings of Miller and Bartick [33], who reported that Raman spectra of nylon fibers collected between 200 and 2000 cm^−1^ (extended to 2400 cm^−1^ only for acrylics) show no significant features in the 2000–2700 cm^−1^ region, thus confirming the absence of fundamental vibrations around 2500 cm^−1^.

Raman spectroscopy can be incorporated into a cleaning validation method to enable systematic monitoring and validation of membrane cleaning protocols. This involves collecting spectra both before and after cleaning cycles and comparing key spectral features. Effective cleaning should result in a significant reduction in the baseline and the disappearance or substantial reduction in peaks associated with organic matter and biofilm components, particularly those near 1600, 1100, and 2900 cm^−1^. The implementation of such an approach would provide a rapid, non-destructive means of verifying the efficacy of cleaning-in-place (CIP) procedures.

Finally, it is recommended that future Raman analyses focus on the active layer of the membranes, where fouling most directly impacts membrane performance. To optimize Raman measurements of this thin polyamide layer, careful control of laser power and acquisition settings is required to prevent sample damage while maximizing signal quality. Additionally, spectral subtraction techniques can be applied to remove background signals from the support layer, enhancing the detection of subtle changes in the active layer’s chemistry.

### 3.3. Membrane Surface Characterization by SEM-EDX and Fouling Profiling

To analyze the mechanisms of spatial fouling, scanning electron microscopy combined with energy-dispersive X-ray spectroscopy (SEM-EDX) was conducted on membrane samples taken from three different locations along the length of the membrane. The fouling profile was obtained from the EDX spectra, and a fouling index (FI) was calculated to quantify the severity of fouling.

**Figure 9:**
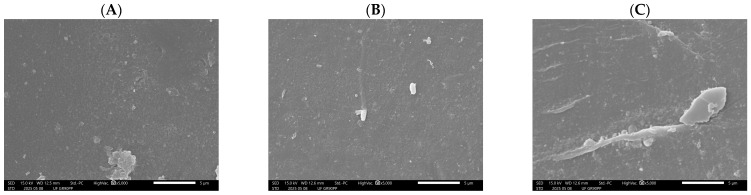
SEM micrographs of UF membrane (GR90PP, 5 kDa) surface after treatment of beet sugar raffinate: (**A**) entrance section, (**B**) middle section, (**C**) end section. Note: The comma in “5,000” is for visual clarity; it represents the number 5000.

The SEM micrographs in Figure 9 show the surface morphology of a UF membrane (GR90PP, 5 kDa MWCO) used for treating by-product stream from the beet sugar industry, taken at three positions along the membrane length: (A) entrance, (B) middle, and (C) end.

Image A (entrance) reveals a relatively clean membrane surface with limited deposition. Only a few scattered particles and a small localized foulant cluster are visible. This suggests that early-stage fouling is minimal and primarily consists of loosely attached particulates or organic residues present in the raffinate.

Image B (middle) shows an increase in surface deposition. Several small particles (including e.g., microorganisms like bacteria) and isolated deposits are now more visible across the membrane surface, indicating the onset of fouling accumulation, likely due to concentration polarization effects that promote the deposition of soluble and colloidal components as the feed progresses along the membrane.

In contrast, Image C (end) displays significant fouling, with a large, dense deposit and evidence of surface coverage and scaling. This pronounced localized fouling at the end of the membrane is consistent with typical trends in pressure-driven membrane systems, where decreased cross-flow velocity and increased concentration polarization toward the membrane outlet enhance fouling and scaling.

The feed solution from beet sugar processing is known to contain a complex mixture of organic matter (polysaccharides, proteins, colorants) and inorganic ions (calcium, magnesium, silica), which likely contribute to the formation of these deposits.

Overall, the SEM results confirm a progressive fouling profile along the membrane length, transitioning from light fouling at the entrance to severe deposition at the outlet. These observations underline the importance of optimizing pretreatment and operating conditions to control fouling in UF applications that are treating challenging industrial streams such as the beet sugar by-product stream.

**Figure 10:**
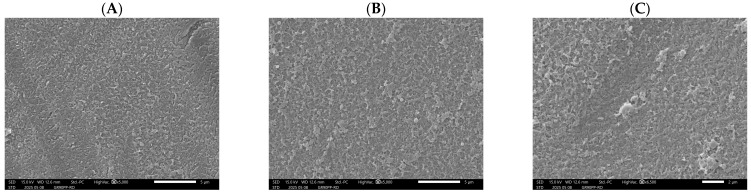
SEM micrographs of RO membrane (RO 98 PHt) surface after treatment of UF permeate derived from beet sugar industry effluent: (**A**) entrance section, (**B**) middle section, (**C**) end section. Note: The comma is for visual clarity.

The SEM micrographs in Figure 10 show the surface morphology of the RO membrane (RO 98PHt) after processing the permeate stream from an upstream UF membrane treating beet sugar raffinate. The images capture three distinct positions along the membrane: (A) the entrance, (B) the middle, and (C) the end.

Image A (entrance of the RO 98PHt membrane) reveals a relatively smooth and clean surface, with only minor textural features and no significant fouling layers. This confirms that the UF pre-treatment effectively removed larger particulates, colloids, and high-molecular-weight organics, resulting in low initial fouling at the RO membrane entrance. Image B (middle) shows a slight increase in surface roughness and the presence of small, evenly distributed deposits. This pattern is indicative of early-stage fouling, likely caused by residual low-molecular-weight organics, salts, or fine colloidal material that passed through the UF membrane and began to accumulate due to concentration polarization effects in the RO process.

Image C (end) displays a more pronounced fouling layer, with noticeable surface roughening and localized deposits. The observed morphology suggests the formation of inorganic scaling and/or bio-organic fouling near the outlet of the RO membrane, where solute concentration and osmotic pressure are highest. This is a common phenomenon in RO systems, particularly when treating complex industrial streams, as the solubility limits of salts can be exceeded at the membrane end, leading to precipitation and scaling.

Overall, the SEM results demonstrate a progressive fouling trend along the RO membrane, though the severity of fouling is relatively moderate, likely due to the beneficial effect of upstream UF pre-treatment. The data highlight the importance of maintaining effective pre-treatment and considering antiscalant dosing or optimized cleaning protocols to mitigate downstream scaling and preserve reverse osmosis (RO) membrane performance during long-term operation.

### 3.4. Fouling Index (FI) and Operational Implications

**Figure 11:**
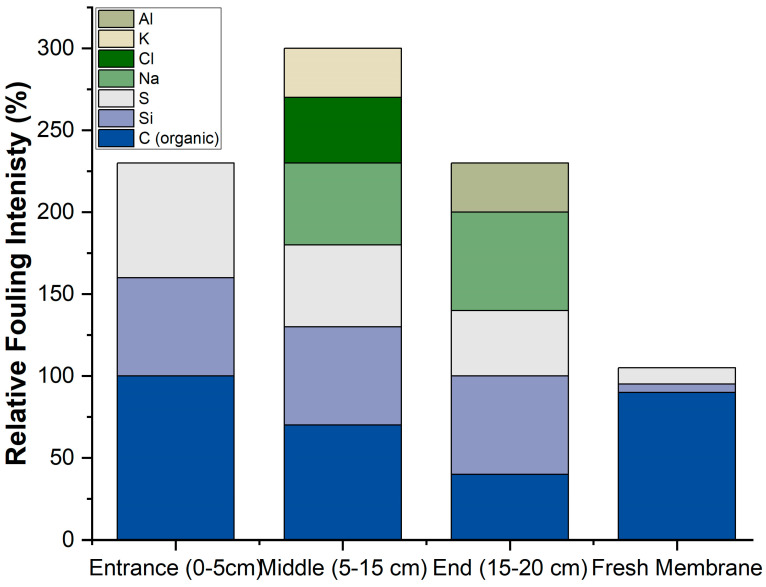
Longitudinal fouling profile along RO membrane length based on SEM-EDX analysis.

Figure 11 presents the longitudinal fouling profile of the RO membrane based on SEM-EDX analysis, showing how the type and intensity of fouling vary along the membrane length. At the membrane entrance (0–5 cm), fouling is predominantly composed of organic matter (represented by carbon) and silica-based compounds, with smaller contributions from sulfur and sodium.

This is typical, as larger organic molecules and colloidal silica tend to accumulate at the front of the membrane due to size exclusion and initial interactions with the membrane surface. In contrast, the middle section of the membrane (5–15 cm) exhibits the highest overall fouling intensity, with a noticeable increase in inorganic components such as chloride, potassium, and aluminum, alongside continued contributions from organic matter and silica. This pattern suggests that concentration polarization effects are stronger in the middle of the membrane, leading to localized precipitation and scaling of ionic species.

Finally, at the membrane end (15–20 cm), the overall fouling intensity decreases, but there is a significant presence of aluminum, indicating possible localized precipitation of alumina or related compounds. Organic fouling at this point is minimal, likely due to either the depletion of organic material along the flow path or flow dynamics that reduce deposition at the end of the membrane.

Table 3 presents the fouling index (FI) and dominant fouling types across different sections of the RO membrane, derived from the sum of normalized EDX intensities for key foulants.

The data reveal a distinct spatial variation in fouling behavior along the membrane length. The entrance section (0–5 cm) exhibits an FI of 230, with fouling primarily attributed to organic matter (carbon and oxygen), accompanied by silica and sulfate scaling. This is characteristic of the initial interaction between feedwater organics and the membrane surface. In contrast, the middle section (5–15 cm) shows the highest FI of 300, indicating a convergence zone where both organic and inorganic foulants accumulate.

Here, mixed fouling is observed, consisting of organics alongside sodium, chloride, potassium, silica, and sulfur. This suggests that concentration polarization effects are most pronounced in this region, promoting precipitation and scaling. The outlet section (15–20 cm) sees a return to an FI of 230, dominated by inorganic scaling—particularly sodium, silica, and aluminum deposits—while organic fouling diminishes.

Operationally, these findings highlight the importance of optimizing pretreatment to reduce organic and colloidal load at the membrane entrance and implementing targeted cleaning strategies to address the combined fouling observed in the middle section. Enhanced antiscalant dosing or pH adjustment may also be necessary to mitigate inorganic scaling toward the outlet.

Based on the observed fouling profile, a two-stage cleaning protocol is proposed to effectively address the spatial variation in foulant composition along the membrane. The first stage should involve an alkaline cleaning step (e.g., using sodium hydroxide-based solutions) to remove organic fouling predominantly accumulated at the membrane entrance. This should be followed by an acid cleaning stage (e.g., using citric acid or hydrochloric acid-based solutions) to target and dissolve inorganic scales, which are more prevalent in the downstream sections of the membrane.

In addition, the application of silica-specific antiscalants and the optimization of upstream ultrafiltration (UF) pre-treatment processes are recommended to mitigate the formation of silica-based scaling and reduce the overall fouling load on the membrane in future operations.

It is recommended that future work focus on evaluating the long-term performance of the integrated UF–RO system during continuous operation for at least 1,000 h. Key parameters, including permeate flux, salt rejection, and fouling indices, should be monitored every 24 h. Additionally, the integrity of the membranes should be assessed through periodic scanning electron microscopy (SEM) imaging and tensile strength testing. The effectiveness of cleaning procedures should be measured by evaluating flux recovery following standardized chemical cleaning methods. Furthermore, it is essential to analyze the quality of the permeate for trace organics and microbial content. These extended durability studies will yield critical insights into operational stability, guide the optimization of maintenance protocols, and validate the system’s reliability for full-scale industrial deployment

### 3.5. Scale-Up Methodology of the Integrated UF–RO Process: From Bench-Scale Data to Plant-Scale

**Figure 12:**
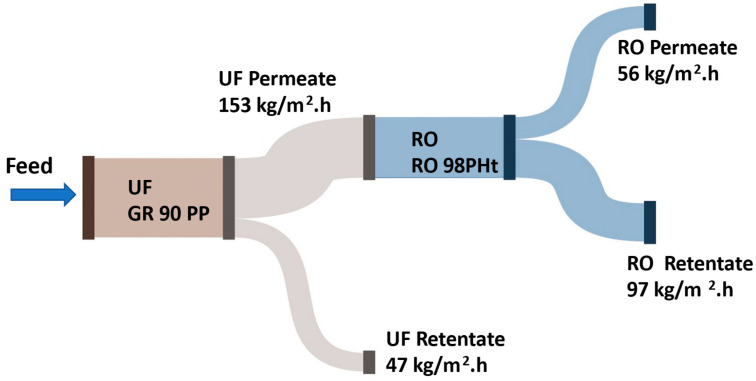
The flux performance of an integrated membrane filtration system.

Figure 12 illustrates the flux performance of an integrated membrane filtration system used for the treatment of raffinate. In this process, a UF membrane, specifically the UF GR 90 PP membrane from Alfa Laval, serves as a pre-treatment stage prior to reverse osmosis (RO) processing. The raffinate feed stream first undergoes UF filtration, which effectively removes suspended solids and high-molecular-weight compounds.

The UF membrane operates with a permeate flux of 153 kg/m^2^·h compared with earlier studies on sugar-beet effluent treatment, the GR90PP ultrafiltration stage achieved a notably higher permeate flux of 153 kg/m^2^·h, versus the 120 kg/m^2^·h reported for ceramic UF membranes clarifying raw beet juice [18], generating a permeate stream that is subsequently directed to the RO system. Meanwhile, the UF retentate is discharged at a flux of 47 kg/m^2^·h. And the water recovery for the UF membrane is 80%.

The permeate from the UF unit, containing dissolved salts and sugars, is then treated using an RO 98PHt membrane, also from Alfa Laval. This RO stage achieves further purification, producing two separate streams: an RO permeate with a flux of 56 kg/m^2^·h compared to roughly 45 kg/m^2^·h observed in sugar-beet press water treatment by Hatziantoniou & Howell [22], representing purified water, and an RO retentate with a flux of 97 kg/m^2^·h, which contains the concentrated solutes. The flux recovery was 93% compared to the recovery flux for direct Ro without treatment, which was 22%.

The sequential combination of UF and RO membranes is designed to optimize the removal of both particulate and dissolved contaminants. The pretreatment role of UF not only improves the efficiency and longevity of the RO membranes but also ensures higher quality permeate, aligning with the objective of obtaining purified water free from salts and sugars.

To convert the bench-scale ultrafiltration-reverse osmosis (UF-RO) cascade into a full-scale treatment process, the first step is to determine the permeation fluxes at the pilot scale: 153 kg/m^2^·h for ultrafiltration (UF) and 56 kg/m^2^·h for reverse osmosis (RO). Additionally, the retentate fluxes are assessed, with values of 47 kg/m^2^·h for UF and 97 kg/m^2^·h for RO. With these values established, fundamental mass balance principles and area-sizing relationships are then applied. For each stage (iii), the required membrane area is calculated by Equation (4).

(4) $$Area=\frac{Permeate\;Flow\;Rate}{Flux}$$

The single-stage recovery is defined as:

(5) $$Recovery\%=\frac{Permeate}{Feed}$$

yielding 76.5% in UF and 36.6% in RO, and the overall recovery in this integrated system is calculated as in the following equation, which is 28.0% recovery.

(6) $$Overal\;Recovery\%=\frac{Permeate\;of\;RO\;stage}{Feed\;of\;UF\;stage}$$

With a target production capacity (e.g., m^3^ h^−1^), these expressions directly yield the total UF and RO membrane areas, which are then arranged in parallel or series–parallel arrays to meet hydraulic-pressure, fouling-control, and cleaning-regimen requirements. The final design incorporates pump-and-pressure-vessel specifications to achieve the necessary transmembrane pressures and crossflow velocities, energy-consumption estimates based on the Watson or Langelier–Lewandowski models, and cleaning-in-place protocols to ensure consistent flux over the plant’s operational lifetime.

## 4. Conclusions

The potential of membrane-based processes, particularly the integrated UF and RO approach, in addressing the challenges of wastewater management within the sugar production industry was investigated at lab scale with a native by-product stream from a sugar refinery.

The results demonstrated that pre-treatment with tight UF membranes with a MWCO of 5 kDa significantly improved RO performance, allowing for a remarkable reduction in permeate conductivity with 0.428 mS/cm and stable flux conditions at around 60 kg/m^2^·h, which represents a water yield of 28% based on the feed stream even with a first try—further optimization can be considered.

The fouling along the length of the flat membrane surface during RO membrane (at the entrance, middle, and end) was analyzed by FTIR, RAMAN, and EDX to understand the type and location of fouling. Understanding the type and location of fouling is crucial for developing strategies that mitigate its impact and optimize membrane performance in industrial applications.

Based on the observed fouling profile, a two-stage cleaning protocol is proposed to effectively address the spatial variation in foulant composition along the membrane. The first stage should involve an alkaline cleaning step (e.g., using sodium hydroxide-based solutions) to remove organic fouling predominantly accumulated at the membrane entrance. This should be followed by an acid cleaning stage (e.g., using citric acid or hydrochloric acid-based solutions) to target and dissolve inorganic scales, which are more prevalent in the downstream sections of the membrane.

In addition, the application of silica-specific antiscalants and the optimization of upstream ultrafiltration (UF) pre-treatment processes are recommended to mitigate the formation of silica-based scaling and reduce the overall fouling load on the membrane in future operations.

Overall, the results highlight a shift in fouling mechanisms along the membrane: from organic and colloidal fouling at the entrance to predominantly inorganic scaling in the middle and end sections. This information can be used to optimize pre-treatment processes and cleaning strategies, such as targeting organic removal before the membrane and using antiscalants or pH adjustment to mitigate scaling along the membrane length.

## Figures and Tables

**Table 1:** Feed composition.

Cl^−^ [mg/L]	SO_4_^−^ [mg/L]	Na^+^ [mg/L]	K^+^ [mg/L]	Ca^++^ [mg/L]	Mg^++^ [mg/L]	Raffinose [%]	Saccharose [%]
451.7	1030.3	1694.8	4756.7	13	2.2	0.2	1.31

**Table 2:** Characterization of RO membrane and UF membrane.

Membrane	Manufacture	Material	Max Temp	Max Pressure
UF-GR90PP	Alfa Laval	Polysulfone	75 °C	10 bar
RO-RO98PHt	Alfa Laval	Thin-film composite	75 °C	40 bar

**Table 3:** Fouling index.

Membrane Section	Fouling Index (FI, a.u.)	Dominant Fouling Type
Entrance (0–5 cm)	230	Organic fouling (C, O) with silica and sulfate scaling
Middle (5–15 cm)	300	Mixed fouling (organics + Na, Cl, K, Si, S)
Outlet (15–20 cm)	230	Inorganic scaling (Na, Si, Al)

## Data Availability

The original contributions presented in this study are included in the article. Further inquiries can be directed to the corresponding authors.
